# Supplementary material for: SARS-CoV-2 Infection in Unvaccinated High-Risk Pregnant Women in the Bronx, NY, USA Is Associated with Decreased Apgar Scores and Placental Villous Infarcts
Source: Biomolecules. 2023 Aug 6;13(8):1224. doi: 10.3390/biom13081224 (PMC10452574; doi:10.3390/biom13081224)
Supplement: Supplementary file 1 [file biomolecules-13-01224-s001.zip › biomolecules-2491735-supplementary.pdf]

**Placental Abnormality**	**Criteria for Diagnosis**
Maternal Vascular Malperfusion	
Small for gestational age placenta	Placental weight less than tenth percentile for gestational age
Distal villous hypoplasia	Decreased number of terminal chorionic villi relative to stem chorionic villi, elongated and thin chorionic villi with increased syncytial knots; findings present in lower two-thirds of section, occupying at least 30% of section
Villous congestion	Terminal chorionic villous vessels filled with confluent erythrocytes; finding present in at least 50% of villous parenchyma
Villous infarct	Well-circumscribed area of coagulative necrosis containing ghost villi
Intervillous thrombus	Well-circumscribed area of clotted blood with laminations, displacing chorionic villi
Decidual vasculopathy	Spiral arteries with incomplete remodeling, hypertrophic change, fibrinoid necrosis, thrombosis or atherosis
Inflammatory Responses	
Chorioamnionitis	Fetal membranes containing neutrophils involving chorion and/or amnion
Funisitis	Umbilical cord containing neutrophils involving at least one vessel
